# miR-27a-5p—Abundant Small Extracellular Vesicles Derived From Epimedium-Preconditioned Bone Mesenchymal Stem Cells Stimulate Osteogenesis by Targeting Atg4B-Mediated Autophagy

**DOI:** 10.3389/fcell.2021.642646

**Published:** 2021-09-21

**Authors:** Xiaoyun Li, Rumeng Chen, Yunchuan Li, Panpan Wang, Yan Cui, Li Yang, Xiaofeng Zhu, Ronghua Zhang

**Affiliations:** ^1^College of Pharmacy, Jinan University, Guangzhou, China; ^2^College of Traditional Chinese Medicine Jinan University, Guangzhou, China; ^3^The First Affiliated Hospital of Jinan University, Guangzhou, China; ^4^Cancer Research Institution, Jinan University, Guangzhou, China

**Keywords:** small extracellular vesicle, miR-27a-5p, bone mesenchymal stem cell, osteogenesis, Epimedium

## Abstract

Osteoporosis (OP) is a disease affecting the elderly and is characterized by incremental fractures and bone fragility. Small extracellular vesicles (sEVs) derived from mesenchymal stem cells have been demonstrated to possess potent regeneration potential. In this study, we evaluated the osteogenesis effects of sEVs derived from Epimedium-preconditioned bone mesenchymal stem cells (EPI-sEV) from osteoblasts and ovariectomized (OVX) rats. The underlying mechanism of EPI-sEV-induced osteogenesis was explored by RNA-sequencing and verified by transfection with the corresponding mimic and inhibitor. EPI-sEV stimulated osteogenic differentiation of osteoblasts and moderated both bone mass and microstructure in OVX rats. Sequencing identified a unique enrichment of a set of microRNAs (miRNAs) in EPI-sEV. Overexpression or inhibition *in vitro* demonstrated that the osteogenesis-inducing potential was primarily attributed to miR-27a-5p, one of the most abundant miRNAs in the EPI-sEV fraction. Dual-luciferase reporter assays showed that miR-27a-5p promoted osteogenesis through direct suppression of Atg4B by targeting its 3′ untranslated region. Additional experiments showed that miR-27a-5p suppressed autophagy that was activated in OVX rats. Moreover, osteogenic differentiation was ablated by the intervention with rapamycin in osteoblasts. These data report the regenerative potential of EPI-sEV to induce osteogenic differentiation of osteoblast cells leading to bone formation. This process is achieved by delivering sEV-miR-27a-5p to target Atg4B for further autophagy stimulation.

## Introduction

Osteoporosis (OP) is a disease that affects the elderly. The onset of OP is initiated by an age-related decrease in hormones, such as estrogen. It has been reported that approximately 50% of postmenopausal women worldwide are affected by OP. The prevalence of bone fractures among osteoporotic patients has been reported as high as 40% (Rachner et al., 2011). Therefore, it is of great importance to ascertain the pathological mechanisms leading to OP to formulate effective therapeutic strategies. It has been demonstrated that transplantation of mesenchymal stem cells (MSCs) exhibits therapeutic effects in several models of disease, including the promotion of osteogenesis in a stabilized fracture (Macias et al., 2020). Therefore, MSCs therapy may provide a new perspective for OP prevention and treatment.

Recently, the effects of MSCs therapy have been attributed to paracrine secretion (Liang et al., 2014). In this therapy, small extracellular vesicles (sEVs), including exosomes, are important components of the paracrine secretion of cells. The sEVs are released into the extracellular space through fusion with the plasma membrane (Mianehsaz et al., 2019). Such vesicles participate in the transport of biochemicals, including cytokines, mRNAs, microRNAs (miRNAs), and proteins, and, as a result, play an essential role in intercellular communication. SEVs containing miRNAs have been highlighted due to their diverse biological functions (Xie et al., 2017). Numerous studies have identified that specific functional miRNAs can be selectively packaged into sEVs, which in turn serve as regulators of bone mesenchymal stem cells (BMSCs). Osteoclast-secreted miR-214-enriched exosomes are transferred to osteoblasts, selectively inhibiting their activity (Sun et al., 2016). Likewise, exosomes from C2C12 myoblasts enhance osteogenic differentiation of MC3T3-E1 by delivering miR-27a-3p (Xu et al., 2018). Therefore, miRNA compromised by sEVs may play a crucial role in the process of bone metabolism.

Epimedium (EPI) is a traditional Chinese medicine that has been used to prevent and treat OP for many years (He et al., 2019). Clinical trials have reported positive effects on bone health, suggesting that compounds or extracts of EPI have the potential to be developed as agents, either alone or in combination with other drugs. These reports indicate that EPI has the ability to prevent or delay the onset of OP and reduce the risk of hip fractures (Indran et al., 2016). Previous studies from our laboratory have shown that a water extract of EPI could moderate bone mass and alter the microstructure of bone tissue in ovariectomized (OVX) rats. The mechanism of how EPI regulates osteocyte activity, in particular how it influences bone metabolism, has been explained in various ways. However, whether EPI influences sEV function, thus altering the bone microenvironment, is still unclear.

Previous studies have demonstrated that the production and bioactivity of sEVs could be highly modulated under diverse physiological and pathological conditions. These indicated that alternations of the cell microenvironment could modulate exosome cargos (He et al., 2018). In this study, we evaluated the effects of sEVs derived from Epimedium-preconditioned BMSCs (EPI-sEV) on osteoblasts and OVX rats. In addition, the miRNA profile in exosomes derived from different treatments of BMSCs was analyzed. The mechanism of miR-27a-5p on osteogenic differentiation was explored in order to ascertain the relationship between EPI, sEVs miRNA, and osteogenesis. Findings from this study may provide new perspectives in the treatment and prevention of bone disease.

## Materials and Methods

### Chemical Components Analysis of the Water Extract of EPI

The chemical constituents of the water extract of EPI were analyzed by high performance liquid chromatography (HPLC). The separation conditions were following our previous study (Liu et al., 2018). Briefly, the mobile phase consisted of a mixture of methanol (A) and formic acid (B). A linear gradient elution was performed under the following conditions: 0–45 min at 10–100% A and 45–60 min at 100% B. Chromatographic peaks were identified according to the retention time, and the UV spectrum data were compared with reference standards.

### Cells Culture and Treatment

Bone mesenchymal stem cells were obtained from Cyagen Bioscience (Guangzhou, China); primary osteoblasts were harvested from 3-day-old rats. Calvarium was digested by collagenase type I and trypsin in alpha-MEM medium (C12641800BT; Gibco, United States) at 37°C with shaking. The first two digestions (10 and 20 min, respectively) were discarded. Following the final digestion (60 min), cells were centrifuged and resuspended in growth medium. BMSCs and osteoblasts were cultured in alpha-MEM medium, containing 10% fetal bovine serum (FBS500-S; Ausgenex Pty, Australia).

The co-culture system: osteoblasts were seeded onto 6-well plates (2 × 10^5^ cells/insert), while BMSCs were also seeded onto 6-well plates (1 × 10^5^ cells/insert) with a transwell filter (0.4 μm; Jet). Cells were grown to 80% adherence and incubated with GW4869, which is a commonly used pharmacological agent that inhibits sEVs generation (D1692; Sigma-Aldrich, United States), or EPI-sEV for 48 h. Then, total pre-osteoblasts RNA was extracted and analyzed by quantitative (q) PCR.

### Exosome Isolation, Characterization, and Uptake

Bone mesenchymal stem cells were plated in 10 cm culture dishes and grown to 80% confluence. The culture medium was replaced with exosome-free serum medium containing 1 μg/ml EPI or not, and then incubated for 48 h. The culture medium was collected and centrifuged at 300 × *g* for 10 min, then 3,000 × *g* for 10 min, followed by 10,000 × *g* for 30 min, and then 100,000 × *g* for 1 h twice. The supernatant was discarded, and the pellet was resuspended with PBS for the following experiment or stored at −80°C. The exosome sample included two groups: one was derived from EPI-sEV, and the other was derived from untreated bone mesenchymal stem cells (Un-sEV).

Exosome concentration was evaluated using a BCA assay kit (23225; Thermo Fisher Scientific, United States). The morphological feature of sEVs was observed by transmission electron microscopy (TEM) (Hitachi, Japan). Briefly, exosome suspensions were mixed with 4% paraformaldehyde and deposited on formvar-carbon-coated electron microscope grids. Digital images were taken. The distribution of size was determined by Nanoparticle Tracking Analysis (NTA) using the Nanosight NS500 (Malvern Instruments, Amesbury, United Kingdom). Briefly, samples were diluted and injected into the sample chamber and then analyzed by the NTA. sEV surface marker proteins were detected by Western blot analysis.

### Cell Viability and Osteogenic Differentiation Assay

Cell viability was examined using the Cell Counting Kit 8 (CCK8) according to the manufacturer’s instructions (CK04; Dojindo, Japan). Osteogenic differentiation was evaluated by the alkaline phosphatase (ALP) assay, Alizarin Red S staining, and ALP staining. The ALP assay and staining kits were purchased from Nanjing Jiancheng Bioengineering Institute (G1480; Beijing, China). Alizarin Red S was obtained from Solarbio Life Science (A059-2-2; Nanjing, China). Reagents were used following the manufacturer’s instructions. Briefly, cells were stained by discarding the culture medium, washed with PBS three times, then fixed with 4% paraformaldehyde, and stained with the above stains.

### Animals and Treatment

Approval for animal care and experiments was obtained from the Ethics Committee of the Experiment Animal Central of Jinan University. Bilateral OVX surgery was conducted on 3-month-old female Sprague–Dawley rats according to the method of Liu et al. (2018) Four weeks after surgery, rats were randomly divided into three groups (*n* = 6 for each group), and tail vein injections were conducted as follows for a total of 12 weeks: a sham control group (sham—sham surgery with phosphate buffer saline treatment), an ovariectomy control group (OVX—ovariectomy surgery with phosphate buffer saline treatment), and EPI-sEV intervened group (EPI-sEV—ovariectomy surgery with 100 ng/ml). Rats were sacrificed, and tissue samples were collected.

### Dual-Energy X-ray Absorptiometry and Micro-CT Analysis

The bone mineral density (BMD) of rats was analyzed by dual-energy X-ray absorptiometry (General Electric Company, United States). Data were assessed by Lunar_iDXA iDXA software intended for small animals to assess bone density, as well as bone mineral content (BMC), fat content, and body mass index (BMI).

The trabecular micro-architecture of the femur and lumbar vertebrae was evaluated by a Hiscan XM Micro CT (Suzhou Hiscan Information Technology Co., Ltd.). The X-ray tube settings were 60 kV and 133 μA, and images were acquired at 50 μm resolution. A 0.5° rotation step through a 360° angular range with a 50-ms exposure per step was used. The images were reconstructed with Hiscan Reconstruct software (version 3.0; Suzhou Hiscan Information Technology Co., Ltd.) and analyzed with Hiscan Analyzer software. Then, the bone volume/tissue volume (BV/TV), trabecular number (Tb.N), trabecular thickness (Tb.Th), and trabecular separation (Th.Sp) data were collected.

### Hematoxylin Staining, Eosin Staining, and Immunohistochemistry

Samples taken from the femur and lumbar vertebrae were fixed with 4% paraformaldehyde, decalcified with 18% EDTA, dehydrated, and embedded with paraffin. Finally, 10 μm thick sections were placed on glass slides and stained with hematoxylin and eosin (H&E). Furthermore, the paraffin sections were used in immunohistochemistry (IHC) experiments. The 10 μm thick sections were prepared, stained, counterstained, dehydrated, hyalinized, and mounted. The antibody dilution for IHC was 1:100. All of the images were captured using a microscope (Zeiss AXIO).

### Western Blot and Immunofluorescence

Total protein was extracted by RIPA lysis buffer, and the concentration of total protein was measured by the BCA protein determination kit (23225; Thermo Fisher, United States). Total protein was separated by SDS-PAGE gel electrophoresis, transferred to PVDF membranes, and then blocked with 5% skim milk. Membranes were incubated with the following primary antibodies (1:1,000) overnight at 4°C: anti-ALP (GTX42809; GeneTex, United States), anti-COL1A1 (E8F4L; Cell Signaling Technology, United States), anti-Runx2 (D1H7; Cell Signaling Technology, United States), anti-BMP-2 (ab225898; Abcam, United States), anti-LC3 (ab192890; Abcam, United States), anti-p62 (ab109012; Abcam, United States), anti-beclin1 (ab62557; Abcam, United States), and anti-GAPDH (D16H11; Cell Signaling Technology, United States) as the internal reference. The following day, membranes were incubated with anti-rabbit IgG secondary antibody (1:3,000, 7074P2; Cell Signaling Technology, United States). Immunoreactive bands were visualized with an ultrasignal chemiluminescent reagent (4AW012; 4A Biotech, China). The results were quantified using ImageJ (NIH, Bethesda, MD, United States).

Immunofluorescence analysis was conducted according to the manufacturer’s instructions (P0183; Beyotime, China). Briefly, discarding the culture medium and fixing cells with 95% ethanol, samples were washed three times for 15 min with TBSTx and blocked with 5% BSA. Samples were then incubated with BMP-2 (ab225898; Abcam, United States) antibodies overnight at 4°C. Thereafter, 4′,6-diamidino-2-phenylindole (DAPI, C1005; Beyotime) was used to stain nuclei before capturing images. The images were captured by microscope (Zeiss AXIO).

### ELISA Assay and Automatic Biochemical Analyzer Examination

Molecular markers, including estradiol (E_2_), parathyroid hormone (PTH), serum type I collagen cross-linked to C-terminal peptide (CTX-1), and bone gla protein (BGP), were measured using commercial ELISA assay kits (Elabscience, China).

The content of calcium and phosphorus in serum was evaluated by an automatic biochemical analyzer (AU5800; Beckman, United States).

### RNA-Sequencing and Real-Time Polymerase Chain Reaction

The exosomes were collected with or without EPI intervention. Total RNA of exosomes was extracted by a miRNeasy Micro Kit (217004; Qiagen, Germany), and the quantity and quality were assayed by the Nanodrop 2000 (Thermo Fisher, United States). Isolated small miRNAs were reverse transcribed into cDNA and amplified according to the manufacturer’s instruction (638313; Takara, Japan). Raw data from small miRNA were obtained using Illumina. The *p*-value and fold change were calculated for each miRNA. Differentially enriched miRNAs were filtered with the standard fold change of ≥2.0 and an FDR value of <0.05. TargetScan and miRbase were applied to predict the target gene of miRNA.

The total RNA was extracted by Trizol reagent, and cDNA was synthesized using a reverse transcriptase kit (820A; Takara, Japan). Next, 40 PCR cycles were performed by ABI 7500 (Life Science, United States). The formula 2^–ΔΔCt^ was carried out to calculate the relative expression of the target gene. mRNA expression was standardized to glyceraldehyde-3-phosphate dehydrogenase. The gene primer sequences are listed in Table 1.

**TABLE 1:** The gene primer sequences of the qPCR.

Gene name		5′–3′ sequence
*Bmp-2*	Forward: Reverse:	GCCATCGAGGAACTTTCAGA TGTTCCCGAAAAATCTGGAG
*Alp*	Forward: Reverse:	GACAAGAAGCCCTTCACAGC ACTGGGCCTGGTAGTTGTTG
*Col1a1*	Forward: Reverse:	ACGTCCTGGTGAAGTTGGTC TCCAGCAATACCCTGAGGTC
*Runx2*	Forward: Reverse:	AACAGCAGCAGCAGCAGCAG GCACGGAGCACAGGAAGTTGG
*Atg4b*	Forward:	ACTTCAGTGTCCTCAACGCTTTCC
	Reverse:	TGCCTTCGCCAACTCCCATTTG
*Lc3*	Forward:	GAGCGAGTTGGTCAAGATCATCCG
	Reverse:	GATGTCAGCGATGGGTGTGGATAC
*P62*	Forward:	AAGTTCCAGCACAGGCACAGAAG
	Reverse:	TCCCACCGACTCCAAGGCTATC
*Beclin1*	Forward:	TCAAGATCCTGGACCGAGTGACC
	Reverse:	TCCTGGCTCTCTCCTGGTTTCG
*Gapdh*	Forward:	GACATGCCGCCTGGAGAAAC
	Reverse:	AGCCCAGGATGCCCTTTAGT

### miRNA Mimic Transfection and Luciferase Reporter Genes

Cells were plated in 6-well plates and grown to 50–60% confluence. The transfection mix, Lipofectamine 3000 (L300008; Invitrogen, United States), was used with the miRNA mimic or inhibitor (Ruibo, China) at 37°C for 24 h. The specific processes were conducted according to a previous study (Li X. Y. et al., 2019). The transfection efficiency of mimic or inhibitor was confirmed by qPCR.

The Atg4B 3′ UTR region was amplified and cloned into the firefly reported vector (Gene Matrix, China). The mutant version of the Atg4B 3′ UTR reporter plasmid was also constructed (Gene Matrix, China).

Cells were co-incubated with 50 nM of the miRNA mimic, 100 nM of the miRNA inhibitor, and the luciferase reporter vector. The Dual-Glo luciferase assay system (E1910; Promega, United States) was used to evaluate the luciferase activity after 48 h post-transfection. Normalized firefly luciferase activity was compared between different groups.

### Statistical Analysis

The data involved in this experiment were reported as mean ± standard deviation and analyzed by the GraphPad Prism 8.0 software. Multiple-group comparisons were evaluated by one-way analysis of variance (ANOVA) with the Newman–Keuls test. *p* < 0.05 was considered to indicate significant difference.

## Results

### The Effect of EPI on Osteogenic Differentiation in BMSCs

**FIGURE 1:**
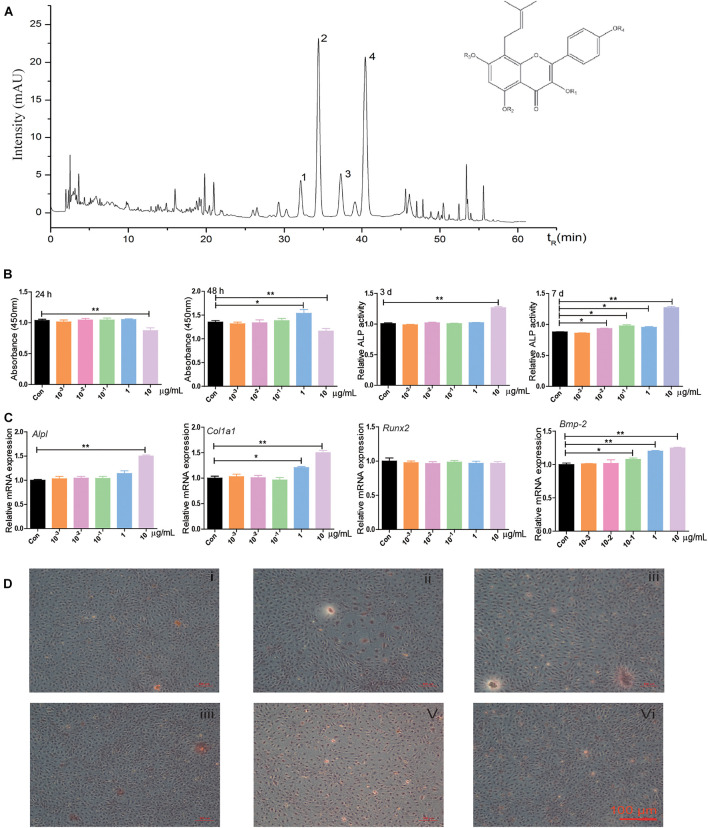
The chemical composition of the water extract of EPI and its effects on proliferation and osteogenic differentiation in BMSCs. **(A)** The HPLC results showed that Epimedin A, Epimedin B, Icariin, and Epimedin C were identified in the water extract of EPI, 1: Epimedin A, 2: Epimedin B, 3: Icariin, 4: Epimedin C. **(B)** The effect of different EPI concentrations (10^–3^, 10^–2^, 10^–1^, 1, and 10 μg/ml) of EPI on BMSCs proliferation and osteogenic differentiation; the raw data represented mean ± SD. **(C)** The mRNA expression of osteogenic differentiation-related factors with different EPI concentrations (10^–3^, 10^–2^, 10^–1^, 1, and 10 μg/ml) of EPI. **(D)** Alizarin Red S staining of BMSCs with different concentrations (10^–3^, 10^–2^, 10^–1^, 1, and 10 μg/ml) of EPI; scale bar: 100 μm. *p < 0.05, **p < 0.01; analyses were done twice and obtained comparable results.

Qualitative analysis of the chemical composition of the water extract of EPI was performed using HPLC. Epimedin A, Epimedin B, Icariin, and Epimedin C were identified (Figure 1A). Further experiments were carried out to evaluate the proliferation and osteogenesis effects of EPI on BMSCs. CCK8 assay revealed that 10 μg/ml EPI inhibited cell proliferation at 24 or 48 h compared with the control group. However, 1 μg/ml EPI stimulated BMSCs proliferation at 48 h (Figure 1B). The 10 μg/ml EPI significantly increased the ALP activity on the 3rd or 7th day, while 10^–2^, 10^–1^, and 1 μg/ml EPI significantly stimulated ALP secretion on day 7 (Figure 1C). Furthermore, 10 μg/ml EPI significantly promoted the mRNA expression of *Alpl* and *Col1a1*, and 1 μg/ml EPI significantly promoted *Col1a1* expression. Alizarin Red S staining showed that the number of mineralization nodules increased when using 1 μg/ml EPI (Figure 1D). Therefore, 1 μg/ml EPI was selected for use in the following experiments based on these results.

### EPI-sEV Stimulates the Osteogenic Differentiation in Osteoblasts

**FIGURE 2:**
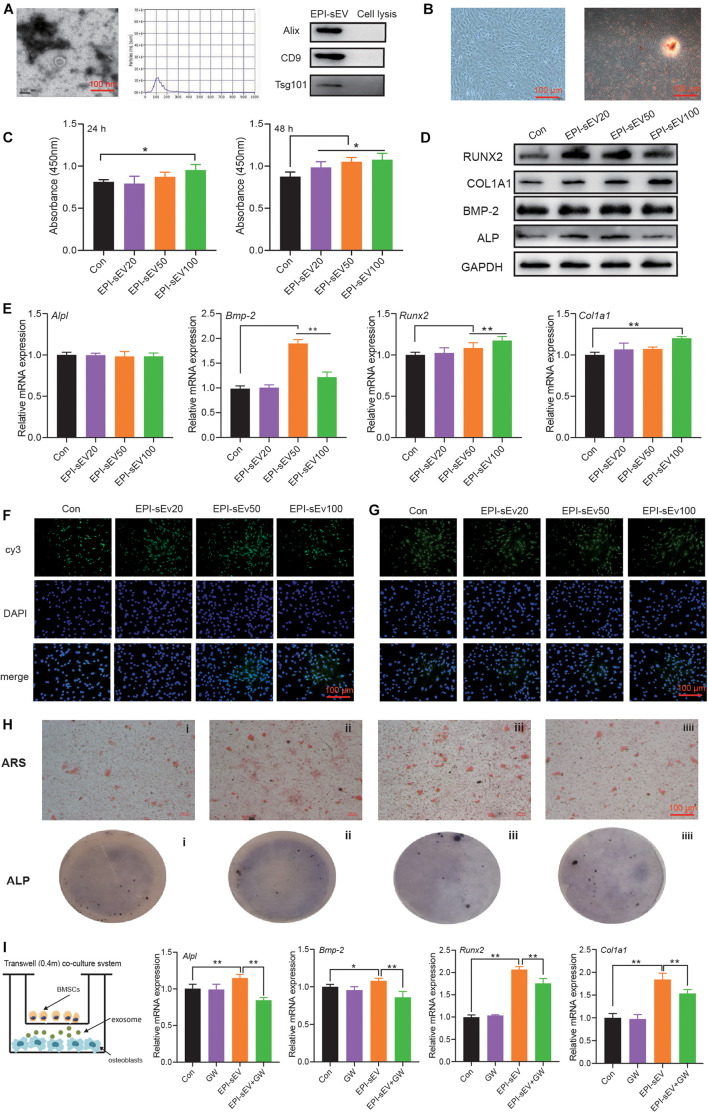
Identification of EPI-sEV and the effect of EPI-sEV on osteogenic differentiation in osteoblasts. **(A)** Characterization of EPI-sEV using TEM, NTA, and Western blot; scale bar: 100 nm. **(B)** Identification of osteoblasts using morphology observation and Alizarin Red S staining; scale bar: 100 μm. **(C)** The effects of different EPI-sEV concentrations on osteoblasts proliferation. Un-sEV: sEVs derived from untreated BMSCs; EPI-sEV20: 20 g/L EPI-sEV, EPI-sEV50: 50 g/L EPI-sEV, and EPI-sEV100: 100 g/L EPI-sEV. **(D)** The protein expression of osteogenic differentiation-related factor with different concentrations of EPI-sEV. **(E)** The mRNA expression of osteogenic differentiation-related factor with different concentrations of EPI-sEV; the normalized data represented mean ± SD. un-sEV: sEVs derived from untreated BMSCs; EPI-sEV20: 20 g/L EPI-sEV, EPI-sEV50: 50 g/L EPI-sEV, and EPI-sEV100: 100 g/L EPI-sEV. **(F)** The immunofluorescence results of ALP and RUNX2 with different concentrations of EPI-sEV. un-sEV: sEVs derived from untreated BMSCs; EPI-sEV20: 20 g/L EPI-sEV, EPI-sEV50: 50 g/L EPI-sEV, and EPI-sEV100: 100 g/L EPI-sEV. **(G)** ARS staining with different concentrations of EPI-sEV. A: Un-sEV group, B: EPI-sEV20, C: EPI-sEV50, and D: EPI-sEV100. **(H)** ALP staining with different concentrations of EPI-sEV. A: Un-sEV group (sEVs derived from untreated BMSCs), B: EPI-sEV20 (20 g/L EPI-sEV), C: EPI-sEV50 (50 g/L EPI-sEV), D: EPI-sEV100 (100 g/L EPI-sEV); scale bar: 100 μm. **(I)** The co-culture system of BMSCs and osteoblasts with GW4869 or EPI; the normalized data represented mean ± SD. *p < 0.05, **p < 0.01; analyses were performed twice and obtained comparable results.

Based on TEM results, sEVs had a typical cup-like appearance with a double membrane structure ranging from 50 to 200 nm. Western blot results indicated that the specific markers of Alix, Tsg 101, and CD9 were highly expressed in the obtained sEV samples; this further described the characteristics of sEVs (Figure 2A).

The cells exhibited typical characteristics of osteoblasts, including a multi-tangle, elongated shape; Alizarin Red S staining revealed various mineralization nodules (Figure 2B). First, we detected the content of EPI in the EPI-sEV to exclude the possibility that those effects were initiated by EPI. There was no detected peak in the HPLC chromatogram (Supplementary Material 1). Second, the osteogenesis effects of EPI-sEV were evaluated by different experiments. The CCK8 results showed that 10 μg/μl EPI-sEV stimulated the proliferation of osteoblasts in 24 h, while the concentration-dependent promotion of osteoblasts proliferation occurred in 48 h, when compared with the Un-sEV group (Figure 2C). The expression of osteogenic differentiation-related factors also increased at different levels with the treatment of EPI-sEV, including Runx2, COL1A1, BMP-2, and ALP (Figures 2D–F). Furthermore, ARS and ALP staining showed that the mineralization nodules and mineral deposits increased with the treatment of EPI-sEV (Figures 2G,H). While co-cultured with GW4869, an inhibitor of sEVs secretion, the osteogenic differentiation of EPI was attenuated (Figure 2I).

### EPI-sEV Enhances Bone Mass and Restores Microstructure in OVX Rats

**FIGURE 3:**
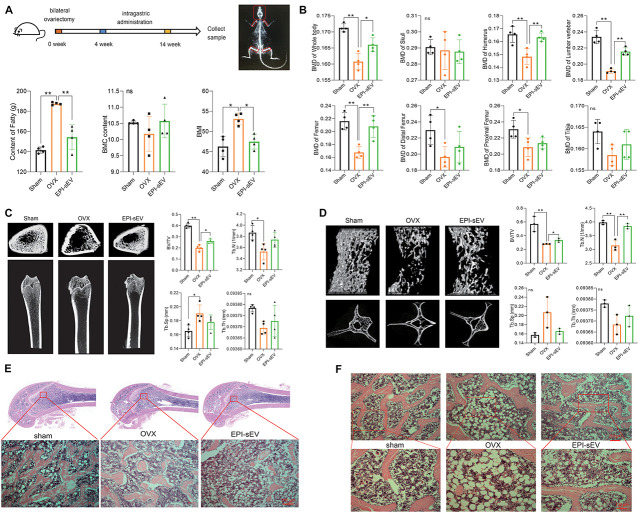
EPI-sEV stimulates bone formation and microstructure restoration in OVX rats. **(A)** The BMC, BMI, and the content of fat in the different groups (*n* = 4); the raw data represented the replicate value. **(B)** The BMD of different positions in the different groups (*n* = 4); the raw data represented the replicate value. **(C)** Micro-CT image and quantitative CT analysis in the distal femur (*n* = 4); the raw data represented the replicate value. **(D)** Micro-CT image and quantitative CT analysis in the lumbar vertebrae (*n* = 3); the raw data represented the replicate value. **(E)** HE staining in the femur; scale bar: 200 μm. **(F)** HE staining in the lumbar vertebrae; scale bar: 200 or 100 μm. *p < 0.05, **p < 0.01; ns, non-significant; analyses were performed twice and obtained comparable results.

In the above experiments, it was demonstrated that EPI-sEV has more potential for osteogenesis than Un-sEV. To further explore the effect of EPI-sEV on OP in rats *in vivo*, OVX rats were administered with EPI-sEV *via* tail vein injection. The results revealed that BMC showed no significant difference between the sham and OVX groups, as well between the OVX and EPI-sEV groups. The content of fat and BMI was compared between the different groups. Compared with the sham group, the results showed that the content of fat and BMI was higher in the OVX group, while those two parameters in the EPI-sEV group were lower than those in the OVX group (Figure 3A). The BMD showed no significant difference in the skull and tibia of the OVX group compared with the sham group, while other positions showed a lower BMD. Compared with the OVX group, the BMDs of the whole body, humerus, lumbar vertebrae, and femur were dramatically increased with EPI-sEV intervention, while the other positions increased without significance (Figure 3B). The femur trabecular BV/TV was higher in the EPI-sEV-treated group than in the OVX group; the value of Tb.N and Tb.Sp increased but without significant differences (Figure 3C). In addition, the micro-CT parameters of the lumbar vertebrae were consistent with the femur, except for the Tb.N, which was higher in the EPI-sEV group than in the OVX group (Figure 3D). The HE staining showed that the trabecular bone appeared thinner, irregular and discontinuous, and lost reticular structure in the lumbar vertebrae of the OVX group compared with those in the sham group. The administration of EPI-sEV helped to restore the normal microstructure (Figures 3E,F).

### EPI-sEV Regulates the Expression of Bone Turnover Markers and Promotes Bone Formation in OVX Rats

**FIGURE 4:**
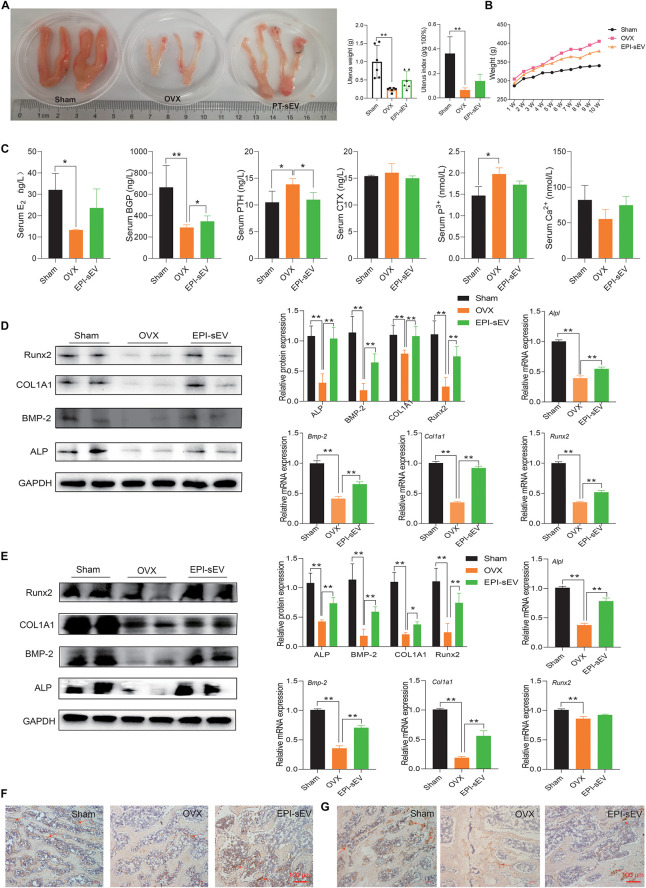
EPI-sEV regulates the expression of bone turnover markers and promotes bone formation in OVX rats. **(A)** The morphology, weight, and index of the uterus in different groups (*n* = 6); the raw data represented the replicate value. **(B)** Variation of weight in different groups (*n* = 6); the raw data represented the replicate value. **(C)** Expression of bone turnover markers in the serum (*n* = 4); the raw data represented the replicate value. **(D)** Protein and mRNA expression of osteogenesis-related factors in the femur; the normalized data represented mean ± SD. **(E,F)** Immunohistochemistry results for COL1A1 in the femur; scale bar: 100 μm. **(G)** Immunohistochemistry results for COL1A1 in the lumbar vertebrae; scale bar: 100 μm. *p < 0.05, **p < 0.01; ns, non-significant; analyses were performed twice and obtained comparable results.

Research indicated that OP is accompanied with the alterations in weight, uterus morphology, and uterus weight. The results showed that the size of the uterus was smaller in the OVX group, while EPI-sEV could moderate this effect. The weight and index of the uterus were lower in the OVX group than in the sham group, while EPI-sEV slightly increased these values in OVX rats (Figure 4A). The rats in the OVX group showed the most significant increase in body weight compared with the sham group, while the EPI-sEV group showed slower growth than the OVX group (Figure 4B). Next, the expression of bone turnover biomarkers was evaluated in the serum, as well the degree of calcium and phosphorous. The results revealed that the levels of E_2_ and BGP were lower, while PTH was higher in the OVX group than in the sham group; with the administration of EPI-sEV, the level of BGP and PTH was slightly restored. The degree of calcium was lower in the OVX group, and EPI-sEV could abate those effects. However, the variation of phosphorous showed an opposite trend (Figure 4C). Then, protein and mRNA expression of bone formation-related factors in the femur and lumbar vertebrae was examined. The results showed that Runx2, COL1A1, BMP-2, and ALP expression was lower in the OVX group than in the sham group. However, those effects were abated in the presence of EPI-sEV (Figures 4D,E). Furthermore, the IHC results showed that the distribution and content of COL1A1 was minimal, while EPI-sEV could attenuate those effects (Figures 4E,F).

### The Specific miRNA Profile in EPI-sEV

**FIGURE 5:**
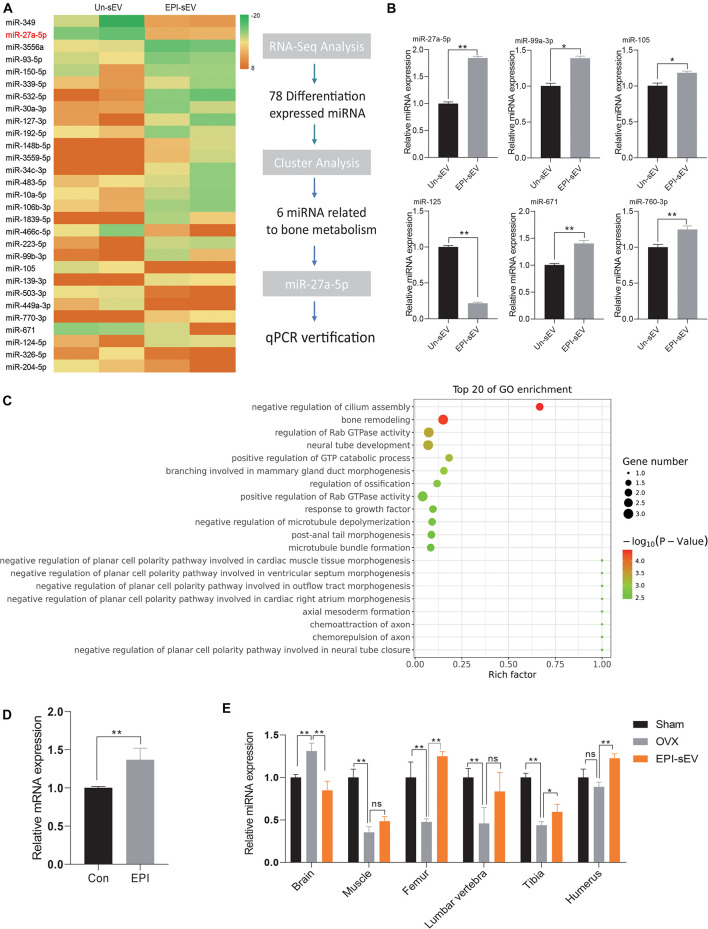
Specific miRNA profiles enriched in EPI-sEV. **(A)** The unique enrichment of a set of miRNAs in EPI-sEV compared with the control group. **(B)** Verification of six miRNAs; the normalized data represented mean ± SD. **(C)** Pathway analysis of the top 20 miRNAs. **(D)** Expression of miR-27-5p expression with or without EPI treatment in BMSCs; the normalized data represented mean ± SD. **(E)** miR-27a-5p expression in different tissues; the normalized data represented mean ± SD. *p < 0.05, **p < 0.01; ns, non-significant; analyses were performed twice and obtained comparable results.

To determine which mechanism was responsible for the prevention of bone loss in OVX rats, the profile of the miRNA in Un-sEV and EPI-sEV was analyzed. The results showed that there are 78 miRNAs differentially expressed in the EPI-sEV group, including 40 downregulated miRNAs and 38 upregulated miRNAs. The top 32 miRNAs are listed in the cluster heat map (Figure 5A). The Gene Ontology (GO) and Kyoto Encyclopedia of Genes and Genomes (KEGG) pathway analyses showed that the differentially expressed miRNAs were closely correlated with bone remodeling and regulation of ossification (Figure 5C). Then, the cluster analysis indicated that there were six miRNAs closely related to bone metabolism (Figure 5B). We used qPCR to identify the accuracy of RNA-Seq. The results were in accordance with the RNA-Seq results. We also detected that the miR-27a-5p expression increased obviously with EPI treatment in BMSCs (Figure 5D). Furthermore, miR-27a-5p expression analysis in different tissues showed that miR-27a-5p expression decreased in the muscle, femur, lumbar vertebra, tibia, and humerus of OVX rats, while with the administration of EPI-sEV, it could significantly increase its expression in the femur, tibia, and humerus (Figure 5E). Therefore, based on the content and variation data, miR-27a-5p was chosen as a potential target for further experiments.

### miR-27a-5p in EPI-sEVs Targets Agt4B-Stimulating Osteogenesis

**FIGURE 6:**
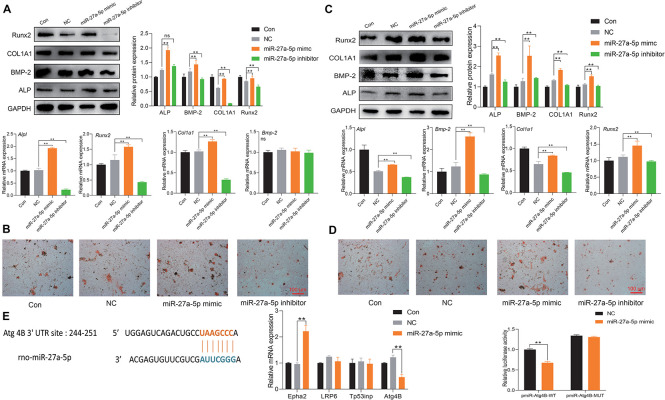
miR-27a-5p targets Agt4B to promote osteogenic differentiation. **(A)** Protein and mRNA expression of osteogenic differentiation-related factor with the intervention of the miR-27a-5p mimic and inhibitor in BMSCs; the normalized data represented mean ± SD. **(B)** Alizarin Red S staining with the miR-27a-5p mimic and inhibitor in BMSCs; scale bar: 100 μm. **(C)** Protein and mRNA expression of osteogenic differentiation-related factor with the miR-27a-5p mimic and inhibitor in osteoblasts; the normalized data represented mean ± SD. **(D)** Alizarin Red S staining with the miR-27a-5p mimic and inhibitor in osteoblasts; scale bar: 100 μm. **(E)** Verification of Atg4B is the target gene of miR-27a-5p. *p < 0.05, **p < 0.01; ns, non-significant; analyses were performed twice and obtained comparable results.

To fully unveil the underlying function of miR-27a-5p, a specific mimic/inhibitor was used to examine the function of miR-27a-5p in BMSCs and osteoblasts. The miR-27a-5p gain and loss experiments in BMSCs or osteoblasts revealed that miR-27a-5p stimulated the protein and mRNA expression of ALP, BMP-2, COL1A1, and Runx2, as well as the formation of mineral nodules (Figures 6A–D). Furthermore, miRBase and TargetScan were used to predict the potential targets of miR-27a-5p, among the predicted target genes. Atg4B was identified as the target gene. Sequence analysis revealed a conserved miR-27a-5p binding site in the 3′ UTR of Atg4B, and the results of qPCR and dual fluorescence report gene indicated that Agt4B is the target gene of miR-27a-5p (Figure 6E).

### Autophagy Is Involved in EPI-sEV-Inducing Osteogenesis

**FIGURE 7:**
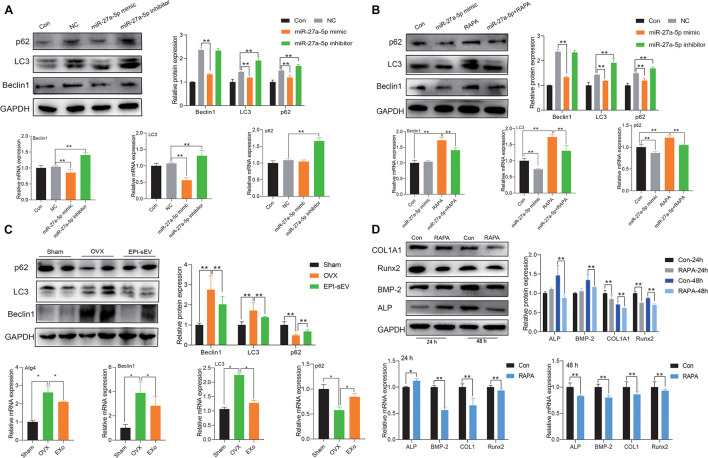
miR-27a-5p-targeted Atg4B mediated autophagy-induced osteogenesis. **(A)** The expression of autophagy-related factor in miR-27a-5p gain and loss experiments in osteoblasts; the normalized data represented mean ± SD. **(B)** Rescue experiments with the treatment of the miR-27a-5p mimic and rapamycin in osteoblasts. **(C)** Expression autophagy-related factor in the OVX and EPI-sEV groups; the normalized data represented mean ± SD. **(D)** Expression of osteogenic-related factor with the treatment of rapamycin for 24 and 48 h in osteoblasts; the normalized data represented mean ± SD. *p < 0.05, **p < 0.01; analyses were performed twice and obtained comparable results.

Atg4B is an important factor involved in the process of autophagy. To further explore the correlation with miR-27a-5p, autophagy, and osteogenesis, the miR-27a-5p mimic and inhibitor were used. The results showed that the protein and mRNA expression of the autophagy-related factor LC3 and Beclin1 was higher, but p62 was lower in the miR-27a-5p mimic group. These effects were attenuated in the miR-27a-5p inhibitor group, when compared with the NC group (Figure 7A). In the rescue experiments, the miR-27a-5p mimic could abate the effects of rapamycin, which activated the autophagy level (Figure 7B). Further study showed that the expression of LC3 and Beclin1 was higher in the OVX group, but p62 was lower in the OVX group than in the sham group (Figure 7C). The ability of osteogenic differentiation was evaluated with rapamycin intervention at 24 or 48 h. The results showed that the expression of osteogenic differentiation-related factor was attenuated in the RAPA group, when compared with the control group (Figure 7D).

## Discussion

Osteoporosis is a serious threat to the quality of life of aging individuals, especially postmenopausal women (Rozenberg et al., 2020). Therapeutic options focus more on suppressing bone absorption and less stimulating bone formation. Nevertheless, the side effects of drug led people to explore new strategies (Chen et al., 2019). EPI is a traditional Chinese medicine showing positive effects on bone remodeling in clinical and pre-clinical research. Various findings indicated that EPI would be a good candidate for the prevention or cure of OP (Indran et al., 2016). In this study, HPLC was used to analyze the chemical constitution of the water extract of EPI. Four compounds were identified: Epimedin A, Epimedin B, Epimedin C, and Icariin. Furthermore, data showed that different concentrations of the water extract of EPI stimulated the osteogenic differentiation of BMSCs. These findings were in accordance with our previous study, as well as other studies (Kim et al., 2017; Yang et al., 2019).

Paracrine secretion of MSCs may be a safer and more promising avenue of OP treatment (Paspaliaris and Kolios, 2019). SEVs, including exosomes, are an important component of the MSCs paracrine responses. Studies have shown that autologous BMSCs, or adipose tissue-derived MSCs, have been used to treat OP (Boeloni et al., 2014). Zhao et al. (2018) demonstrated that exosomes derived from BMSCs stimulated osteoblasts proliferation and prevented OP. In the present study, sEVs evaluated their effects on osteoblasts. The results indicated that EPI-sEV enhanced the proliferation of osteoblasts, stimulating osteogenic differentiation and promoting the mineralization of nodule formation.

In *in vivo* experiments, EPI-sEVs had no significant effects on the uterus, meaning that EPI-sEVs do not exert estrogen-like effects. However, it has been reported that fatty acid oxidation is decreased and the body weight is increased in OVX rats (Paquette et al., 2009; Chen et al., 2018). In the present study, the content of fat, BMI, and body weight was also increased in OVX rats, which could also be moderated by EPI-sEV treatment. The femur and lumbar vertebrae tend to be more fragile and suffer more bone loss than other bones in the body (Sozen et al., 2017). In this study, the BMD was higher in both the femur and lumbar vertebrae with EPI-sEV treatment in OVX rats. The microstructure and quality of the bone trabecular network was also affected. Bone turnover markers are another type of indicator that reflects the status of bone metabolism, including bone formation and destruction indicators (Goltzman, 2018). The results showed that the expression of serum BGP could be raised, while PTH was attenuated by EPI-sEV treatment. Furthermore, the expression of proteins and mRNA of osteogenic-related factor increased with the treatment of EPI-sEVs. Therefore, the above data indicated that EPI-sEVs stimulated the activity of osteoblasts and were also involved in the process of bone reconstruction.

Small extracellular vesicles are an effective intermediate between cell signaling and the action-at-a distance influence on the bioactivity of recipient cells (Yue et al., 2020). Research has shown that the miRNA profiles of sEVs were altered at different osteogenic differentiation stages (Wang et al., 2018). In the present study, we compared the miRNA profiles of BMSC-derived sEVs with or without EPI intervention. The results revealed that there is a series of miRNA expression alternates, including miR-503-3p, miR-204-5p, miR-10a-5p, and miR-27a-5p. GO and KEGG analyses showed that differentially expressed miRNAs are involved in bone remodeling and regulating ossification. Both miR-503 and miR-204 play a role in osteoblast proliferation and apoptosis in response to PBMT (Zhang et al., 2018; Li Q. S. et al., 2019). In addition, miR-27a is essential for osteogenic differentiation in MSCs (You et al., 2016). The mRNA verification experiments indicated that the results of RNA-Seq were accurate. Further miR-27a-5p gain and loss experiments showed that miR-27a-5p promoted osteogenesis. These results were in accordance with the results showing that the expression of miR-27a decreased in osteoporotic patients (Gu et al., 2016) and promoted osteogenesis in steroid-induced rat BMSCs (You et al., 2016). The analysis of bioinformation revealed that the target gene of miR-27a-5p was Atg4B. The results of dual-fluorescence reporter gene and qPCR indicated that Atg4B is the target gene of miR-27a-5p. Hence, our results suggest that miR-27a-5p within EPI-sEVs plays a vital role in the process of sEVs-mediated osteogenesis in BMSCs.

It has been reported that Atg4B is important for the fusion between autophagosomes and lysosomes (Fu et al., 2019), and that it is lacking Atg4B-reduced autophagy (Akin et al., 2014). Therefore, it was investigated whether if miR-27a-5p targeting Atg4B mediated autophagy in the process of osteogenesis. Concomitantly, miR-27a-5p gain and loss experiments revealed that the activation of autophagy was negative relative to the expression of miR-27a-5p. Likewise, rapamycin is an activator of autophagy. Rescue experiments indicated that rapamycin could abate the effects of miR-27a-5p mimic. Furthermore, bone tissue protein and mRNA experiments showed that autophagy was activated in the OVX rats, while the level of autophagy could be attenuated with the intervention of EPI-sEV. Likewise, Li J. et al. (2019) showed that autophagy increased in OVX rats. In the present study, we detected that the expression of osteogenic differentiation-related factor was attenuated with the intervention of RAPA in osteoblasts. Hu et al. (2019) have shown that induced autophagy results in a decline in osteoblast differentiation, and that the level of Atg4B is closely related to the bone resorption (Deselm et al., 2011). Therefore, we demonstrated the regenerative potential of EPI-sEV to induce osteogenic differentiation of osteoblast cells leading to bone formation. This process is achieved by delivering sEV-miR-27a-5p to target Atg4B for further autophagy stimulation, which may provide new perspectives for the treatment and prevention of bone disease. Though the present study showed the efficiency of EPI-sEV in OVX rats, there were also some limitations. The study did not further demonstrate the effects of miR-27a-3p-overexpressed exosome in OVX rats. It also did not investigate the deeper mechanism between autophagy and EPI-sEV. In future study, more evidences will be needed to support this conclusion.

## Conclusion

The present study demonstrated that EPI-sEV stimulated osteogenesis in OVX rats and osteoblasts by delivering miR-27a-5p, which targeted Atg4B-mediated autophagy. EPI-sEV may become an innovation therapeutic method and is proposed to prevent OP in the future.

## Data Availability Statement

The data analyzed in this study is subject to the following licenses/restrictions: The data and materials presented in the study are deposited in the https://www.jianguoyun.com/. Accession number will be given when reasonable request. Requests to access these datasets should be directed to 402574826@qq.com.

## Ethics Statement

The animal study was reviewed and approved by Jinan University.

## Author Contributions

RZ and XZ designed and conducted the whole experiment. XL and RC completed most of the experiments and wrote the manuscript. YL and PW assisted the measurement of related parameters. YC and LY analyzed the data. All authors contributed to the article and approved the submitted version.

## Conflict of Interest

The authors declare that the research was conducted in the absence of any commercial or financial relationships that could be construed as a potential conflict of interest.

## Publisher’s Note

All claims expressed in this article are solely those of the authors and do not necessarily represent those of their affiliated organizations, or those of the publisher, the editors and the reviewers. Any product that may be evaluated in this article, or claim that may be made by its manufacturer, is not guaranteed or endorsed by the publisher.
